# Renouncing the attempt versus perpetration distinction

**DOI:** 10.1007/s11229-022-04000-6

**Published:** 2023-01-11

**Authors:** Izabela Skoczeń

**Affiliations:** Faculty of Law and Administration and Jagiellonian Centre for Law, Language and Philosophy, Jagiellonian University, Krakow, Poland

**Keywords:** Renunciation defense, Moral luck, Legal luck, Responsibility, Moral psychology, Attempt law

## Abstract

Legal and moral luck goes against the basic principle of criminal law that responsibility ascriptions are based on the mental state of the perpetrator, rather than merely the outcome of her action. If outcome should not play a decisive role in responsibility ascriptions, the attempt *versus* perpetration distinction becomes more difficult to justify. One potential justification is that we never know whether the attempter would not have resigned from pursuing her criminal intent even at the last moment. However, this paper argues that resigning from criminal intent and trying to stop the criminal outcome, which is called the renunciation defense, can be just as subject to outcome luck as the attempt *versus* perpetration distinction. And yet the availability of the renunciation defense in court is outcome dependent. I show with a series of experiments (N = 479) that outcome dependence for the renunciation defense is perceived as unjust and discuss the implications for the renunciation defense as well as attempt *versus* perpetration distinction.

**Supplementary Information:**

The online version contains supplementary material available at 10.1007/s11229-022-04000-6.

## Introduction

The present paper argues that the criminal law (in many countries) credits successful, but not unsuccessful, renunciations of criminal action. This practice is analogous to the criminal law's punishing successful attempts (labeled perpetrations) more harshly than unsuccessful attempts. To the extent that this distinction resembles strict liability, it calls out for special justification. As it will be shown with experiments, based on empirical findings, lay people don't attribute a meaningful distinction between unsuccessful and successful renunciations. This empirical fact provides prima facie evidence that the law should not treat the cases differently. This is especially pertinent as the renunciation case is a special case of the classical attempt *versus* perpetration debate. This is because, an agent that commits a completed attempt but undertakes renunciation of the criminal action before the outcome occurs is morally better than an agent that merely completes an attempt. However, the empirical studies presented in the paper can also shed new light on traditional arguments in the debate justifying the attempt *versus* perpetration distinction. Namely, one potential justification of the mentioned distinction is that we never know whether the attempter would not have resigned from pursuing her criminal intent even at the last moment. However, this paper argues that resigning from criminal intent and trying to stop the criminal outcome, which is called the renunciation defense, can be just as subject to outcome luck as the attempt *versus* perpetration distinction. The paper shows with a series of experiments (N = 479) that this outcome dependence is perceived as unjust, which undermines the classical argument supporting the attempt *versus* perpetration distinction.

### The problem of moral luck

Imagine two people speeding on the same road, at the same time and in the exact same circumstances. A child jumps into the way of one of the drivers which results in the child’s death, while the second driver is lucky—no such outcome occurs. If luck is to influence moral responsibility, then the child-killer is more responsible than the ‘mere’ speeder. Yet is it fair to treat differently two people that took the same risk and the same decision? A negative answer entails endorsement of the ‘control principle’. This is the claim that responsibility for an action and its consequences is not affected by factors which are not under the control of the actor (Hartman, 2017; Nagel, 1979; Nagel & Williams, 1976). In other words, we should ideally be responsible only for actions which are under our control. This however begs the question on how to define ‘control’ as such. A prominent idea is defining control through comparison of relevant contexts (counterfactual reasoning). This idea is labeled the ‘comparative control principle’: if persons S1 and S2 are exactly alike with respect to some event X, except regarding factors that are external to each person’s agency, then S1 and S2 are equally responsible with respect to X (for an overview cf. Hartman, 2017).

### Legal luck versus strict liability

In terms of legal responsibility, both drivers will most probably face rather different consequences: the child-killer will be detained, while the “mere” speeder will get away with a fine. As the drivers’ case shows, when luck influences moral responsibility it often simultaneously influences legal responsibility. “Legal luck” obtains where one’s legal status – such as legal rights, obligations, liabilities, and culpability – turns on facts not under one’s control (Ripstein, 2001; Enoch, 2007, p. 28; Green, 2017; Herstein, forthcoming). The theoretical debate on legal luck concentrates either on negligence cases (Feinberg, 1962; Waldron, 1997; Schroeder, 1997; Herstein, forthcoming) or on the law of criminal attempts (Feinberg, 1962; Davis, 1986; Lewis, 1999; Kessler-Ferzan, 1994; Kadish, 1994; Alexander et al., 2009).

Luck makes responsibility dependent largely on the outcome of one’s actions, irrespective of with which mental state one acted. This is close to what lawyers call ‘strict liability’. Strict liability requires negligence, however the principal probative requirement is that the agent caused the outcome, rather than that she acted with a certain mental state (Waldron, 1997). To appreciate the distinction, I will refer to the view that solely outcome determines responsibility as the ‘consequence-oriented approach’.

### The controversies surrounding strict liability

Criminal law is by and large against strict liability and advocates that culpability should be determined on the basis of the mental state of the perpetrator irrespective of whether her actions produced any consequences. This means that, if two agents took the same decision, then they should be held equally culpable.

Robert Thomas writes that ‘the law has long recognized a presumption against criminal strict liability—the Supreme Court describes it as a "generally disfavored status"'’ (Thomas, 2012, p. 649).^1^ The Supreme Court of the United States stated that:

The contention that an injury can amount to a crime only when inflicted by intention is no provincial or transient notion. It is as universal and persistent in mature systems of law as belief in freedom of the human will and a consequent ability and duty of the normal individual to choose between good and evil.^2^

The American Model Penal Code in its Sect. 2.02 states that the requirement for culpability is that a person acted with the mental state (either purpose, knowledge, recklessness or negligence) required by the Code. If the mental state (so called *mens rea*) is absent, then no culpability ensues. Analogously, the German Criminal Law Code states in its Sect. 15 that: unless the law expressly provides for criminal liability for negligent conduct, only intentional conduct attracts criminal liability. The French Criminal Code also expresses the necessity of the required mental state of at least negligence in its article 121-3. These three codifications have influenced a number of other legal systems around the globe.

Thus, the relevant state of the mind (at least negligence) is required for culpability within criminal law. According to the rules of the cited codes, if there is no relevant mental state, then no culpability ensues within criminal law, a view hereby advocated as ‘Kant-oriented’ as it is a consequence of adopting a Kantian approach to criminal law (Kant, 2009). By default, the ‘consequence-oriented’ element in strict liability in the criminal law is unwelcome and any judgment which does not take into account the mental state of the perpetrator necessitates a special reason.

Moreover, even in tort law, a system designed mainly to compensate damages, which contains several rules imposing strict liability,^3^ it is controversial what is the justification for these strict liability rules. Jules Coleman argues that it is rather the ‘fault system’ that should be applied in tort law. The fault system is defined as attributing responsibility on the basis of fault rather than the action’s outcome, where fault is understood as unreasonable behavior for which one is blameworthy (Coleman, 2002; Kneer, 2021). He writes that ‘even if the fault system is not required by considerations of justice, the question remains whether it is nevertheless morally superior to the rule of strict liability’ (Coleman, 2002, p. 265). According to Coleman strict liability in tort law is problematic when it attributes responsibility for behavior, which is not blameworthy: ‘the traditional view is that strict liability is an unjust theory of responsibility in torts because it does not allow the defense of freedom from fault to defeat an attribution of liability.’ (Coleman, 2002, p. 285).

Nevertheless, a partial exception to the criminal law’s general distaste for strict liability can be found in the way that the so-called renunciation defense is granted to perpetrators in practice, which currently makes room for strict liability.

### The renunciation defense

“In the evening of August 10, 1979, Smith, and his uncle, Melvin Howell, were heavily engaged in a drinking bout in New Albany, Floyd County, Indiana. Though they had never had trouble before, for obscure reasons they fell to quarreling. While the parties dispute which one was the aggressor, it is admitted that Smith stabbed his uncle in the chest twice. The uncle then fled up the street, pursued by Smith, the latter shouting unintelligible epithets at his uncle as they ran. The uncle collapsed from weakness a block away and when Smith approached, his mood had changed. *He was remorseful and wept. Smith then dragged Uncle Melvin into the uncle's car, threw away the knife, "floored the accelerator," and sped up Pearl Street, his destination being the Floyd Memorial Hospital.* At this point a policeman, Corporal Victor Steward, becomes the chronicler of events. He observed Smith going north on Bono Road where Smith's car crashed into a parked automobile. Stewart, with lights flashing and siren wailing, went in pursuit of the speeding Smith who, upon disentangling his car from the parked car, had accelerated at high speed, with lights out, down Conner Street. Smith finally arrived at the hospital without further recorded incident. Examination by physicians at the hospital revealed that *Uncle Melvin had suffered two deep stab wounds between his ribs and close to his heart which had penetrated and collapsed both lungs. Astonishingly, Uncle Melvin survived his wounds and his transportation to the hospital and recovered (…)*” (*State vs Smith*, Indiana 1980).

Smith was really lucky. Since Uncle Melvin miraculously survived the collapse of both his lungs, Smith was able to apply for a substantial mitigation of punishment because he merely attempted the crime as well as did everything he could to prevent Uncle Melvin from dying. However, had Uncle Melvin succumbed to the wounds, which was indeed highly probable, Smith would be in an incomparably worse position—according to the American Model Penal Code he could face 7 additional years in prison or even life imprisonment. It is striking that whether Smith’s genuine remorse is taken into account depends on sheer luck. After all, it was pure luck that the murder failed to occur. Strikingly, Smith’s legal assessment would be almost identical in every American State (except Arizona and Hawaii).^4^

The American Model Penal Code (MPC) qualifies Smith’s and Uncle Melvin’s case as a ‘completed attempt’ (art 5.01.1.b) because Smith stabbed Uncle Melvin twice in the chest causing a very high probability of death.

Article 5.01.4 MPC defines the defense of renunciation as a possibility of punishment mitigation for complete attempts if the perpetrator has actively repented the criminal result. “The defense [of renunciation] is allowed even where the last proximate act has occurred, but the criminal result can be avoided e.g., where the fuse has been lit but can still be stamped out.” This means that renunciation is possible even in the case of a completed attempt (Model Penal Code § 5.01, Comment, p. 72, Tent. Draft No. 10, 1960; Yaffe, 2010).

Renunciation “usually contains specific subjective requirements, such as a complete and voluntary renunciation.” (Robinson, 2018) ‘Complete’ means that the crime commitment cannot be merely postponed but has to be abandoned. ‘Voluntary’ means that it cannot be motivated by increased difficulty or possibility of detection (Robinson, 2018). The MPC shaped the legal rules on renunciation in twenty-six American jurisdictions since it was released (Tsen Lee, 1997). Most jurisdictions require simply that the defendant have “prevented the offense.” A minority of jurisdictions (i.e. Arizona, title 13-1005 of the Arizona Revised Statutes and Hawaii, HI Rev Stat § 705-530 (2017)) require only that the defendant have made “*reasonable* efforts to prevent the offense,” regardless of whether his efforts were in fact successful (Moriarty, 1989; Robinson, 2018).

Since in cases of already completed attempts the success in stopping the criminal effect as a result of second thoughts is often a matter of (causal) luck, the liability equally relies on luck.

Thus, in the cases I will analyze, the success of renunciation determines whether the crime is completed or attempted. Consequently, what is the crux of the problem, is the attempt *versus* perpetration distinction, while success in renunciation is just one factor that is employed to distinguish the two.

In the lucky case (successful renunciation), the perpetrator can employ the renunciation defense. This can result either in reduced criminal liability, or, even in no criminal liability at all. This is because the lucky perpetrator is held responsible for a merely attempted, rather than committed, crime. By contrast, the unlucky perpetrator will be denied a renunciation defense in most systems and will face responsibility for crime commitment. This is confirmed by case law in both lucky cases^5^ and unlucky cases.^6^

Analogous legal rules are present in France (121-5 of the French Criminal Code) or Germany (art 24 of the German Penal Code).^7^ In the United Kingdom, the defense is well established for complicity law and less common for ‘simple’ attempts, yet mentioned as a possibility in the literature (Ashworth & Horder, 2013). Moreover, Antony Duff, advocates the availability of the renunciation defense only when successful repentance occurs (Ashworth & Horder, 2013; Duff, 1996). In Poland (art 15 of the Polish Penal Code) there is a possibility of punishment mitigation for unsuccessful renunciation inscribed in the legal rule, yet the case law demonstrates a reverse trend—unsuccessful renunciation is rarely taken into account (cf. V KK 406/16 and II AKa 65/16).^8^

As depicted in Table 1, the availability of the renunciation defense, only in successful active repentance cases, is independent of whether the system is a continental or common law system: the USA (cf. 23rd rule of the Federal Rules of Criminal Procedure), the UK (Juries Act 1974), as well as a civil law country with lay juries like Japan, all have analogous regulations. In France or Germany the decision lies usually in the hands of professional judges and still the legal rules are similar.^9^

**Table 1:** The availability of the renunciation defense, depending on outcome luck as well as the availability of jury trial in different countries

	France	Germany	Poland	Switzerland	UK	USA
Successful renunciation	Punishment mitigation	Punishment mitigation	No punishment	Punishment mitigation	Punishment mitigation	Punishment mitigation
Unsuccessful renunciation	Perpetration	Perpetration	Perpetration + mitigation (but contrary case law)	Perpetration	Perpetration	Perpetration (+ mitigation in Arizona and Hawaii)
Lay juries	Practically no	Practically no	Practically no	No	Yes	Yes

Thus, when renunciation is unsuccessful, the legal assessment ignores the active repentance undertaken by the accused. This leaves one with a strikingly consequence-oriented^10^ picture based solely on factual causal chains: if the renunciation is not successful, then the accused is charged for perpetration. If it is successful, then the accused is at most charged for attempt or escapes criminal responsibility as she can use the renunciation defense. Thus, in both, successful and unsuccessful, renunciation cases the deeds of the protagonist are identical. However, in the unsuccessful case, the presence of severe outcome changes the assessment of the action of the agent. This is striking if the occurrence of the outcome depends merely on luck, as it introduces a strict liability regime. Moreover, after active repentance, the occurrence of the severe outcome is neither under the agent’s control nor within the scope of the agent’s intention.

Thus, if one renounces criminal intent for a completed attempt, it can well be the case that the success of renunciation is merely a matter of luck, while the mental state and action structure of the successful and unsuccessful (in stopping the criminal outcome) agent is the same. Yet, is it just to base criminal responsibility merely on outcome luck which becomes dangerously close to the strict liability regime?^11^

This incites the hypothesis that, perhaps, the current state of affairs concerning the renunciation defense is not the result of a careful, all things considered judgment about what is fair. Rather, it is the result of the way the criminal procedure is constructed. Namely, in common law countries, if lay juries have to decide on cases involving renunciation, they never get to see the relevant counterfactual case. Rather, they are always presented with either the lucky or unlucky perpetrator’s case. Thus, there is never a chance to compare the two outcomes and reflect on the fact that outcomes may be the result of sheer luck. Moreover, jury instructions also contain no incitement to consider alternative outcomes. For example, the Vermont model jury instructions do not contain a regulation concerning completed attempts and renunciation. The Modern Federal Jury Instructions by Leonard B. Sand and John S. Siffert also do not contain a specific instruction on the renunciation defense. However, they do contain a reference to the *United States versus Crowley* case, in which the verdict states that “counsel should be careful about requesting an instruction on the defense because it requires the argument that the defendant ‘began with a criminal purpose but abandoned it.’ The court suggested that it may be a better choice to argue that defendant never had the necessary criminal intent rather than relying on a defense ‘novel in federal law, and perhaps lacking in jury appeal’.

The present paper aims at verifying with survey experiments whether it indeed is the case that participants find that mere outcome of an action needs to influence responsibility, or rather, they do so because they never get a chance to reflectively compare the two possible, alternative outcomes. Perhaps the consequence-oriented formulation of the legal rules is not necessarily the result of a careful, all things considered, consideration of the principles that should determine responsibility for criminal outcomes.

## Luck, morality and the law

### Different responses to the problem of moral luck

The literature on moral luck is incredibly vast and I do not aspire to give a full account of it in this paper, which is limited in scope. The vast literature centers around the question whether luck should play a role in moral judgment.

An argument for a positive answer is the claim that excluding the influence of luck on the assessment of our responsibility means shrinking the scope of human agency to basically nothing. This is because, there is always a sense in which luck influences each and every one of our actions. For example, the character traits with which we are born with are not our choice, they are a matter of character luck. The decisions and circumstances we face are also a matter of luck upon which we have often no control (so called circumstantial luck). Finally, the consequences of our actions can also be a matter of luck (so called outcome luck). If we exclude responsibility ascriptions which are infused by all these kinds of luck, then little is left of our agency and free will (for an overview see Nelkin, 2019; Herstein, forthcoming).

Moreover, moral luck is compatible with a certain kind of voluntariness: ‘the kind of consequence that a person foresees or could reasonably be expected to foresee, because, in an important sense, it is up to the agent whether these kinds of results obtain’ (Hartman, 2017, p. 91). Thus, undertaking a criminal action is like gambling or taking part in a lottery: if the foreseen outcome does not occur, then the agent receives a smaller punishment for an attempted crime, while if the foreseen outcome occurs, then more punishment is inflicted upon the agent for perpetration (Lewis, 1999; Otsuka, 2009). Consequently, according to these claims. it is acceptable that luck influences the attempt *versus* perpetration distinction.

However, there are also claims to the contrary, namely, that luck should not influence responsibility attributions. This is because, it is unfair to hold people responsible for things which are outside of their control and are not foreseen, or could not have been foreseen. Both the attempter and the successful perpetrator take the same decisions and the same risks – their state of the mind is identical. Moreover, the likelihood of the harm occurring is identical in both the good and bad luck cases.^12^ ‘Desert is a function of the actor’s culpability and that culpability is a function of the risks of harm to protected interests that the actor believes he is imposing and his reasons for acting in the face of those risks’ (Alexander et al., 2009).

Additionally, there might be a qualitative difference between luck and the decisions we take: ‘It is not just that we have more control over our choices than over our constitution, our circumstances, and what we cause. Our control over our choices is different in kind, not different in degree. Bad luck before choice and bad luck after choice is just bad luck; unlike choice, it cannot affect our culpability’ (Alexander et al., 2009, pp. 190–191).

### Moral psychology supports anti-luck

The literature on moral luck, which makes claims on how our responsibility attributions should be (normative claims) is also replete with descriptive claims about what the folk thinks about moral luck. Namely, the claim about the Difference Intuition (DI), which states that the folk assesses the lucky and unlucky agents differently. This intuition is explicitly expressed in numerous papers, let us present a couple of examples. Already Thomas Nagel writes in his seminal paper that:

if the condition of control is consistently applied, it threatens to erode most of the moral assessments *we find it natural to make* (Nagel, 1979).

Dana Nelkin mentions in her Stanford Encyclopedia of Philosophy entry on moral luck that:

Upon reflection, it *seems* that we morally assess people differently for what they do (or who they are) when their actions and personal qualities depend on luck of all kinds (Nelkin, 2019).

Moreover, David Enoch and Andrei Marmor in their paper discuss the case of two drunk drivers (Brian and Arnold) driving in the same circumstances, Brian killing a pedestrian while Arnold being lucky. They conclude the example in the following:

For it *does seem* like a robust intuition, one we too would be loathe to discard, that Brian should feel this special kind of regret, the kind that Arnold need not, and that if Brian does not have these feelings he is morally worse for that (Enoch & Marmor, 2007, p. 15).

Later the authors write that:

when we condemn someone for what he is, we *seem* to be making a moral judgment about things that are out of his control, things that just happen to be as they are, purely a matter of luck (Enoch & Marmor, 2007, p. 25).

However, the plausibility of the philosophical arguments in favor of moral luck based on intuitions about the folk’s assessment might need a close scrutiny from the empirical perspective. This is because, in every-day contexts, we are rarely presented with both, lucky and unlucky, cases at once. Thus, it is not clear whether the difference intuition is a careful, all-things-considered judgement about moral responsibility or rather, a quick and unreflective decision driven by affective reactions toward the observed harm.^13^

Moral psychology shows with survey experiments that, when assessing intentional action, people are much more prone to blame when a bad outcome occurs than to praise when a good outcome occurs, which is called the Knobe effect (Knobe, 2003). Additionally, the more severe the outcome, the more blame is ascribed, which is the so called severity effect (Kneer & Bourgeois-Gironde, 2017; Frisch et al., 2021; Kneer et al., in preparation; Garcia Olier & Kneer, 2022).

Moreover, as studies show, when presented with a vignette describing only either the good or bad luck scenario of negligent behavior (between-subjects design), participants assess the responsibility of the protagonists differently, in accordance with the Difference Intuition, (cf. Cushman, 2008; Kneer & Machery, 2019; Kneer & Skoczeń, 2021; Nichols et al., 2014; Spranca et al., 1991; Young et al., 2010). By contrast, when presented with both scenarios at once (within-subjects design), the Difference Intuition vanishes (Kneer & Machery, 2019; Kneer & Skoczeń, 2021).

Thus, perhaps the Difference Intuition about criminal legal responsibility is the effect of an unreflective judgment while a careful, analytical reconsideration of the matter leads to unfairness intuitions. Moreover, perhaps the Difference Intuition is the result of the unavailability of the good luck alternative outcome in the courtroom. Finally, the increased responsibility (and blame) attributions in the bad luck scenario can be the result of the so-called hindsight bias (cf. Fischoff, 1980; Próchnicki et al., 2022; Teichman, 2014). This means that when presented with the bad outcome, participants retrospectively think that it must have been more likely that the outcome would have occurred and so the agent deserves more responsibility (Kneer & Skoczeń, 2023).

To sum up, contrary to philosophers’ theoretical guesses, experiments show that, when reflective, the folk does not endorse the Difference Intuition and thus believes that outcome luck should not influence our responsibility attributions.

### Motivating the inquiry

If one agrees that moral and legal luck should not influence responsibility ascriptions also for intentional crimes, then consequently one accepts that the attempt *versus* perpetration distinction makes little sense. However, there is one more argument that speaks in favor of preserving the attempt *versus* perpetration distinction, even if one thinks that outcome luck should not influence responsibility attributions. This is the claim that we can never be sure whether the attempter would not have resigned from her criminal intentions even at the very last moment (Brink, 2013; Yaffe, 2010). However, I think that this argument is not fully accurate.^14^

This is because, as argued in Sect. 1.4, renunciation is subject to resultant luck just as much as the perpetration *versus* attempt distinction. In other words, it is possible to conceive of a case in which one resigns from criminal intent at the last moment and yet the outcome occurs. These cases are well possible. Moreover, the pragmatic argument that if you try hard enough you can stop the criminal outcome is irrelevant for luck cases, in which it is pure luck, rather than effort, which is decisive.

Thus, upon reflection, there is little reason to deny moral significance of renunciation in cases where the bad outcome occurs. In other words, there is a moral difference between an agent that, does everything she can to stop the criminal outcome from occurring and yet does not succeed due to bad luck; and an agent who attempts the crime but fails due to luck (for example, a sudden gust of wind changes the direction of the bullet cf. Husak, 2011). Yet currently, the construction of the renunciation defense makes it the case that criminal outcome blocks any significance of renunciation, and this seems unjust.

As argued in Sect. 1.4, for instance Polish law allows for admitting the significance of renunciation in the unlucky case, yet case law shows that this rule is rarely applied. So is it really that folk morality views renunciation differently? Or rather, is it the case that, just as with responsibility attributions in negligence cases, the current application of the renunciation defense is the result of a quick, unreflective judgment, rather than a careful, reflective assessment of responsibility^15^? Let’s check with a survey.

Finally, it is true that criminal law also has the function to deter potential future offenders from bringing about criminal outcomes such as death. This pragmatic deterrence function could constitute a potential explanation of the luck-infused construction of the renunciation defense (Brink, 2013; Duff, 2003; Yaffe, 2010). However, this argument does not work in tort law, in which the deterrence function is much less prominent (Thomas, 2012). Nevertheless, as argued in Sect. 1.3, in tort law strict liability is also controversial, as the fault principle is the morally better alternative for a responsibility basis. Thus, in tort law, renunciation assessment might be most vulnerable to outcome luck.

## Study 1 (between subjects)

### Participants

I recruited 271 lay participants by providing them with a link to the survey via the online platform Amazon Mechanical Turk. The IP address location was restricted to the USA. Participants who failed an attention check, were not native speakers of the English language or took less than 15 s to answer the first question were excluded, leaving a sample of 217 participants (50% of participants were female; their mean age was 40 years with a standard deviation of 12 years, the age range was 19–73 years).^16^

### Methods and materials

Having passed an attention check, participants were presented with the following vignette involving renunciation:

Anna, a botanist, wants to poison a tree in her neighbor’s garden because the tree obstructs her view. During the night, she injects acid into the soil surrounding the tree. After a few days, Anna has second thoughts. At night, she injects the soil surrounding the tree with an alkali solution, which, at a recent botanists' conference, was discussed as an effective antidote to acid poisoning of plants.

Next, participants were randomly assigned one of the two endings (labels in bold omitted):

**Good luck:** The alkali solution works perfectly and the tree remains healthy.

**Bad luck:** The alkali solution does not work and the tree dies.

After reading the ending, participants were asked to answer three moral judgment questions on a 1–7 Likert scale:

1. How wrong were Anna’s actions? (1 = not wrong at all; 7 = extremely wrong)
2. To what extent is Anna blameworthy for her actions? (1 = not at all blameworthy; 7 = extremely blameworthy)
3. How much punishment does Anna deserve for her actions? (1 = no punishment at all; 7 = very severe punishment)

The three questions were followed by an abstract scenario:

Suppose two people A and B each poison a tree. When doing so, A and B are in the same frame of mind, use the exact same poison, and administer it in the same way. The poison takes two days to become active.

Suppose, however, that both A and B have second thoughts, and provide the poisoned trees with an antidote. The antidote is exactly the same in both cases and has a very high likelihood of completely cancelling out the poison’s negative effects. Now imagine that even though the circumstances are exactly the same in the two situations, the antidote administered by A works: the tree is entirely unharmed. But it fails to work in the case of B, and so the poisoned tree dies.

Thereafter, participants were asked to rate how much they agreed with the following three statements (on a 1–7 Likert scale, with 1 anchored at ‘completely disagree’ and 7 anchored at ‘completely agree’):

1. The actions of A are exactly as wrong as those of person B.
2. Both A and B are equally blameworthy.
3. Both A and B deserve an equal amount of punishment.

Finally, participants answered a demographic questionnaire.

### Results

Participants answered in accordance with the difference intuition: a mixed-design ANOVA determined that, aggregating across the three dependent variables (wrongness, blame and punishment), participants judged the action of the agent in the bad luck scenario to be worse than the action of the morally lucky agent (*F* (1,248) = 79.95, *p* < .001, η^2^ = .075). Furthermore, the analysis revealed that, aggregating across the two good and bad luck conditions, the difference in judgment type (wrongness, blame and punishment) was significant (*F* (2,496) = 406.50, *p* < .001, η^2^ = .621).

Bonferroni-corrected post hoc tests showed that the only significant differences were between the punishment question as well as the 2 remaining dependent variables (wrongness and blame) corrected: p < .001, η^2^ = .621. I did not find any evidence that participants responded differently to the other pairs of questions (blame and wrongness: corrected *p* = .103). These the two main effects were qualified by an interaction (*F* (2,496) = 25.24, *p* < .001, η2 = .092). To analyze this interaction, I compared the answers on the questions for each dependent variable respectively (wrongness, blame and punishment) between the two conditions: good *versus* bad luck. The results are summarized in Table 2.

**Table 2:** Effect of outcome on wrongness, blame, and punishment judgments in a between-subjects design; 95% confidence intervals are given for the means

	Between subjects design			
	*T*	*p*	*Cohen’s d*	95% CI
Wrongness	− .57	.568	.08	[− .51; .28]
Blame	− 4.76	< .001	.62	[− 1.19; − .45]
Punishment	− 6.28	< .001	.79	[− 1.58; − .83]

The results of the abstract comparative task can be found in appendix section A.1.2.2.

## Study 2 (within subjects)

### Participants

I recruited 88 lay participants via the same online platform Amazon Mechanical Turk. The IP address location was again restricted to the USA. Participants who failed the attention check, did not indicate the English language as their mother tongue or took less than 15 seconds to answer the first question were excluded, leaving a sample of 73 participants (54% of these participants were female; their mean age was 37 years with a standard deviation of 11 years, and an age range between 19 and 70 years). I preregistered jointly studies 1 and 2.^17^

### Methods and materials

Study 2 was identical in all respects to Study 1, except that participants were presented with *both* vignettes at once (renunciation good and bad luck in randomized order). In the bad luck scenario, the protagonist name was switched to Carol. Next, participants had to judge *both* Anna’s and Carol’s actions separately in terms of all three dependent variables (wrongness, blame and punishment). The wrongness questions, for instance, read “How wrong were Anna's and Carol's actions?”. Participants had to rate Anna’s action, and next Carol’s action, on separate Likert scales ranging from 1 (“not wrong at all”) to 7 (“extremely wrong”).

The questions were followed by the same abstract task as for Study 1.

### Results

**Fig. 1:**
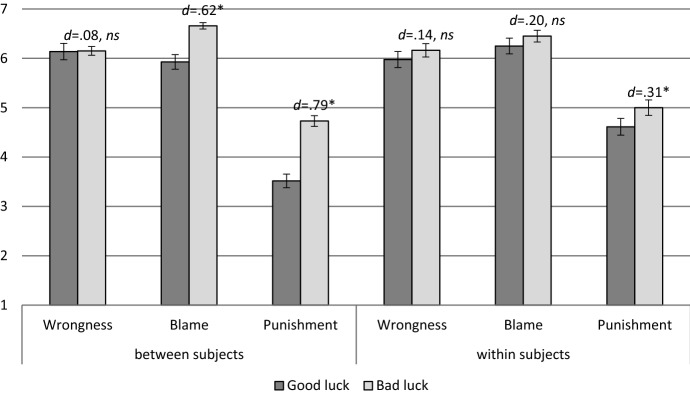
Mean wrongness, blame, and punishment judgments for the between-subjects (Study 1, left panel) and withi-subjects designs (Study 2, right panel); effect sizes are given in terms of Cohen’s d, significance is reported at the p < .050 threshold; error bars denote standard error of the mean

To check whether the outcome – good or bad luck – influenced participants’ answers, I analyzed the answers by means of a two-way (Moral Luck: lucky vs. unlucky; Judgment Type: wrongness vs. blame vs. punishment) repeated-measures ANOVA. I found that aggregating across the three judgment types, participants’ mean responses for the good luck condition differed significantly from the bad luck condition (F (1,79) = 42.31, p < .001, η^2^ = .349; Fig. 1).

I also found that, aggregating across the two moral luck conditions, participants’ mean answers to the wrongness, blame, and punishment questions differed significantly (F(2,158) = 61.66, p < .001, η^2^ = .438). Bonferroni-corrected post hoc tests depicted that there were significant differences between answers to all moral questions: all p < .001.

Importantly, the two main effects I observed were qualified by a two-way interaction (F(2,16) = 46.95, p < .001, η2 = .373). To analyze this interaction, I compared the difference between the morally lucky and the morally unlucky conditions for each of the three questions. I also calculated the effect sizes as well as the confidence intervals for each of these three dependent variables. The results are summarized in Table 3. Crucially, the effect sizes of the difference between the lucky and unlucky conditions in the within-subjects design (2) are cut by more than half in comparison to the between subjects design (1) (with the exception of wrongness, which is less sensitive to outcome in line with the findings in Kneer & Machery, 2019). Moreover, the difference in blame answers across the good and bad luck condition, which was significant in the between subjects’ design, has become insignificant in the within subjects’ design.

**Table 3:** Effect of outcome on wrongness, blame, and punishment judgments in a between-subjects design (Study 1, left panel) and a within-subjects design (Study 2, right panel); 95% confidence intervals are given for the means

	Between subjects design (1)				Within subjects design (2)			
	*t*	*p*	*Cohen’s d*	95% CI	*T*	*p*	*Cohen’s d*	95% CI
Wrongness	− .57	.568	.08	[− .51; .28]	− 1.40	.167	.14	[− .47; − .08]
Blame	− 4.76	< .001	.62	[− 1.09; − .45]	− 1.98	.052	.20	[− .50; .00]
Punishment	− 6.28	< .001	.79	[− 1.58; − .83]	− 3.45	< .001	.31	[− .71; − .19]

#### Proportions

**Fig. 2:**
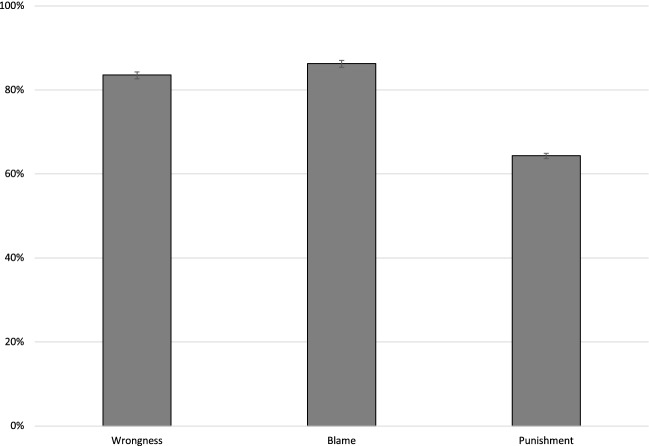
Proportions of participants who judged the lucky and unlucky agents identically (*No DI*) in terms of wrongness, blame, and punishment; errors bars denote 95% confidence intervals; Wilson method, see Brown, Cai, & DasGupta, 2001

Just as Kneer & Machery, 2019, to investigate whether the observed aggregate influence of moral luck on wrongness, blame, and punishment judgments is widespread among participants or is rather the result of the views of a small minority, I compared the proportion of participants manifesting and failing to manifest the Difference Intuition. Participants who judged the lucky and the unlucky agents identically were classified as manifesting “no Difference Intuition” (no DI for short in Fig. 2). The vast majority of participants did not share the Difference Intuition for wrongness (81%), blame (83%), though the proportion was smaller for punishment (64%). The proportions for all dependent variables differed significantly from chance (for wrongness and blame: binomial test p < .001, two-tailed, while for punishment: binominal test p = .018, two-tailed). Punishment judgments differed significantly from wrongness and blame judgments (binomial tests, test proportion = .64, p < .001). Wrongness judgments differed significantly from blame and punishment judgments (binomial test, test proportion = .81, p < .001, all two-tailed). Blame judgments differed significantly from wrongness and punishment judgments (binominal test, test proportion = .83, p < .001).

#### Abstract comparative scenario

**Fig. 3:**
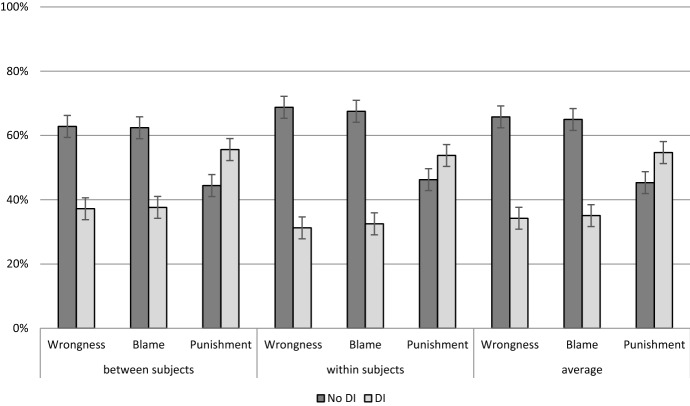
Proportions of participants who chose a 7 on the Likert scale and judged the lucky and unlucky agents identically (*No DI*) or differently (DI) with respect to wrongness, blame, and punishment in the abstract condition errors bars denote 95% confidence intervals; Wilson method, see Brown, Cai, & DasGupta, 2001

In the abstract, comparative scenario, which followed the within-subjects design, 69% of participants answered the wrongness question with a 7 on the Likert scale—they judged the lucky and unlucky actor identically. 68% performed identically with respect to blame and 46% with respect to punishment (cf. Fig. 3).

**Fig. 4:**
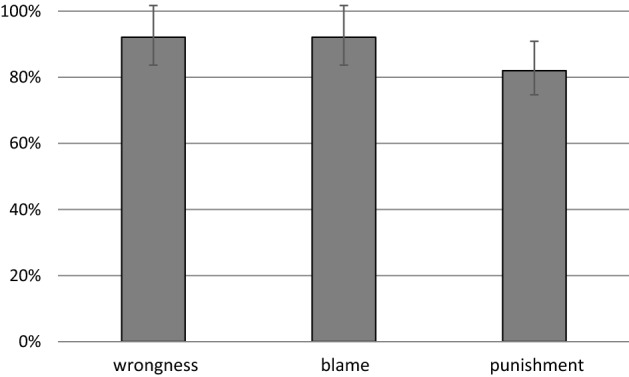
Proportion of participants who agreed with the claim that the two agents should be judged similarly with respect to wrongness, blame, and punishment in the non-abstract scenario (> 4 on the Likert scale). Error bars represent the 95% confidence interval

93% of the participants chose more than the midpoint (4) on the Likert scale with respect to wrongness in the abstract comparative scenario following the within-subjects design. 90% did so with respect to blame and 79% with respect to punishment. More details can be found in the Appendix Section A.1.2.2.

### Discussion

Wrongness appeared to be insensitive to the effect of outcome. By contrast, blame appeared to be sensitive to outcome in the between-subjects design, yet not in the within-subjects design. Finally, punishment revealed to be sensitive to outcome both in the between and within subjects’ designs.

As predicted, the study revealed three patterns in terms of the mean answers on the moral responsibility questions, the outcome effect and the proportions of participants who judged the protagonists identically in both scenarios. First, in the between-subjects design, the mean ascriptions of blame and punishment were higher in the bad luck condition than in the good luck condition. By contrast, wrongness ascriptions remained similar in both the good and bad luck conditions. Moreover, the effect size of the difference in mean ascriptions of blame and punishment across the good and bad luck conditions was more than twice as big in the between-subjects design as compared to the within-subjects design. In other words, when participants saw both conditions simultaneously, they tended to judge the protagonists similarly. The significant difference in blame judgments accros the good and bad luck conditions in the between subjects’ design became insignificant in the within subjects’ design (cf. Kneer & Machery, 2019; Kneer & Skoczeń, 2023; Schwitzgebel & Cushman, 2012; Spranca et al., 1991).

Second, the effect sizes in the within-subjects design were smaller than in the between-subjects design for blame and punishment but not for wrongness. Wrongness appears to be insensitive to outcome in contrast to punishment, which was sensitive to outcome in both between and within subjects’ design. In the within-subjects design, the outcome had a more pronounced effect on mean punishment than on wrongness and blame. This is in line with the findings in (Kneer & Machery, 2019) who observe that blame patterns with wrongness and differs from punishment. However, it is unexpected when compared to (Cushman, 2013; Cushman et al., 2009) who reports that blame patterns with punishment.

Moreover, the effect of outcome in the abstract comparative task was weaker than in the concrete tasks. Additionally, the effect of outcome in the abstract comparative task was much stronger for punishment than for wrongness and blame: contrary to Study 2, most participants judged in the abstract condition that the two protagonists should be punished differently. Third, a considerable proportion of participants assessed wrongness and blame identically in the within-subjects design and in the abstract comparative task. This proportion was significantly lower for punishment. Thus, punishment is the most outcome-sensitive measure of responsibility ascriptions. This is in line with the theory that punishment has a didactic function of changing behavior in future cases (for a detailed model and theory cf. Martin & Cushman, 2016).

## Study 3 (contrastive design)

Study 3 employed a contrastive design to further increase participants’ reflective comparison of the two types of cases.

### Participants

I recruited 120 lay participants again via Amazon Mechanical Turk. I restricted the IP address location to the USA. Participants who failed the attention check, who were not native speakers of the English language or took less than 25 seconds to answer the first question were excluded, leaving a sample of 103 participants (51% of participants were female; the mean age was 40 years with a standard deviation of 12 years; the age range was 21–72 years). The study was preregistered.^18^

### Methods and materials

After passing an attention check, participants were presented with both vignettes just as in Study 2 (the order was fixed: bad luck first). However, the formulation of the wrongness, blame and punishment questions differed from Study 2. In Study 3, participants were presented with statements explicitly comparing both protagonists, Anna and Carol, with respect to each of the three dependent variables:

1. Carol’s actions were just as wrong as Anna’s.
2. Carol is just as blameworthy as Anna for her actions.
3. Carol deserves just as much punishment as Anna for her actions.

Each statement (order randomized) was followed by a 7-point Likert scale anchored at 1 with “completely disagree” and at 7 with “completely agree.” Next, participants were presented with the abstract scenario task.

### Results

A one-way repeated-measures ANOVA determined that participants’ mean answers to the wrongness, blame, and punishment questions differed significantly (F (2,204) = 4.04, p = .019, η2 = .038).

Post hoc tests using the Bonferroni correction revealed that there were significant comparisons between answers to the dependent variables (all corrected p-values above .019). Participants disagreed more with the claim that the morally lucky and morally unlucky agents deserved the same punishment than they disagreed with the claims that their actions were equally wrong and equally blameworthy, and I did not find any evidence that they responded differently to these two latter claims. The means for all three measures were significantly above the neutral midpoint 4 and significantly below the endpoint 7 (complete agreement), see Section A1.2.1. of the Appendix. Analogously, in the abstract comparative task participants’ mean answers to all three questions did differ significantly (cf. Appendix section A 1.2.2).

Following Lench et al., 2015 as well as Kneer & Machery, 2019, I calculated the percentage of participants who agreed with the claim that the two agents should be judged identically. I also aggregated the number of participants who responded with “completely agree” (Likert scale endpoint 7). The results (see Table 4) were consistent with the findings from Study 2: For wrongness and blame the large majority of participants (over 86%) agreed that the two agents should be judged identically (Likert scale > 4). Way over half of the participants chose the endpoint of the Likert scale. Slightly less—82% of the participants agreed that the two agents should be punished the same, and over a half completely agreed with such an assessment, see Fig. 4.

**Table 4:** Proportions of participants who agreed (Likert scale > 4) or completely agreed (endpoint 7) that the two agents should be judged identically with respect to wrongness, blame, and punishment

Measure	Wrongness	Blame	Punishment
Likert Scale > 4	86%	88%	82%
Endpoint 7	70%	71%	59%

### Discussion

Study 3 replicates and extends the findings of Study 2. Participants rather uniformly hold that the two agents should be judged the same in terms of wrongness, blame and, contrary to what (Kneer & Machery, 2019) find, also in terms of punishment. Mean agreement levels differ significantly from the scale’s midpoint; over 80% of the responses are on the “agree” spectrum of the Likert scale and the majority “completely agrees” with an identical assessment in terms of wrongness (70%), blame (71%), and punishment (59%). As in Studies 1 and 2, outcome had a larger impact on punishment judgments than on the other dependent variables, yet still a majority of participants (59%) completely agreed that the actions of the morally lucky and unlucky agents deserve equal punishment. The difference in punishment assessments between the present study and the (Kneer & Machery, 2019) contrastive study might be due to the scenario employing an action which is considered largely less morally condemnable (harming plants rather than people). This points out that perhaps blame and punishment are intertwined – a hypothesis requiring further studies.

According to Studies 2 and 3, which employed a within-subjects and contrastive design respectively, most people do not share the Difference Intuition for wrongness and blame, as well as, according to the contrastive study – also punishment. This again, just as in (Kneer & Machery, 2019) raises the question of whether there is a puzzle of outcome luck in the first place.

Following (Kneer & Machery, 2019 as well as Kneer & Skoczeń, 2023), the between-subjects results from Study 1 consistently replicate the effect of outcome on moral judgment, which constitute an important phenomenon in its own right: in ordinary life, moral and legal judgment resembles the between-subjects design, as we rarely compare actual outcomes to alternative ones.

## General discussion

### Outcome luck and responsibility

Just as in (Kneer & Machery, 2019 as well as Kneer & Skoczeń, 2023) the experiments reveal that outcome has a strong influence on moral judgment. In my studies, I measured the ascriptions of three types of moral judgment variables: wrongness, blame and punishment. I also employed a between-subjects design as well as a within-subjects design and later compared the results.

Just as in (Kneer & Machery, 2019), wrongness ascriptions were the least sensitive to outcome. Blame was sensitive to outcome in the between-subjects design, yet much less in the within subjects’ design (effect sizes reduced by half), which hints toward the bias or inconsistency account (cf. Baron, 2008). Namely, blame ascriptions are not the product of a cautious, all things considered judgment, but rather result from a hindsight bias fueled by the unavailability in courtroom reasoning of the alternative counterfactual (Alicke, 2000; Kneer & Skoczeń, 2023; Lench et al., 2015). Since juries are only presented with either the unlucky or (less often) lucky renunciation case, they fall prey of the unavailability of the alternative scenario in the courtroom proceedings. Finally, in this study, punishment ascriptions were sensitive to outcome both in the between and within subjects design, which points toward the accuracy of Cushman’s dual process model of moral judgment (Cushman et al., 2009). The model predicts roughly that while blame ascriptions are in line with mental states ascriptions (identical mental state, similar blame, even if different outcome), punishment ascriptions are driven by causal judgments. In the bad outcome case, more punishment is ascribed because of the descriptive causal chain between the outcome and the action of the protagonist (irrespective of the protagonist’s mental state).

In the first experiment, in the within-subjects design, the outcome had a more pronounced effect on mean punishment than on wrongness and blame. This is in line with the findings in (Kneer & Machery, 2019) who observe that blame patterns with wrongness and differs from punishment. However, it is unexpected when compared to (Cushman, 2013; Cushman et al., 2009) who report that blame patterns with punishment. Thus, in this respect, these findings are partially inconsistent with the so-called ‘dual pathway model’ developed in (Cushman, 2013; Cushman et al., 2009), similarly as in (Kneer & Skoczeń, 2023).

In the abstract comparative task from Study 1 as well as in the contrastive design from Study 3, a considerable majority of participants assessed wrongness and blame identically, while slightly less assessed punishment identically. I take this as evidence that the difference intuition (differentiating responsibility based on factors outside of the agent’s control) is not as widely held as philosophers postulate and the puzzle of moral luck results from the unavailability of the alternative scenario in most real-life, courtroom judgments. Thus, perhaps careful, reflective judgments of moral and legal responsibility align with the Kant-oriented rather than the consequence-oriented account of what is a fair account of responsibility. In other words, differentiating responsibility on the basis of factors which are not under the agent’s control (for instance outcome) is not considered fair and acceptable by the majority.

### Legal implications: toward renouncing the attempt versus perpetration distinction

In my experiments I tested scenarios where an agent first undertakes a criminal action, while later, due to second thoughts, tries to undo the action. Whether she manages to stop the criminal outcome from occurring is a matter of luck. Moreover, this is a rare case where responsibility is a matter of merely one type of luck, namely outcome luck. No character luck is involved: the agent struggles with herself and changes behavior, which I take as a hint toward indeterminism and control over one’s psychological makeup.

From the legal point of view, in the practice of courts, there exists an asymmetry: one can employ the renunciation defense and ask for punishment mitigation only if one’s actions of renunciation are successful in stopping the criminal outcome. However and problematically, as I argue, just as the attempt *versus* perpetration distinction, this distinction can be a matter of sheer outcome luck. This means that the law adopts a consequence-oriented approach: if the agent is lucky and manages to stop the criminal outcome from occurring, she is to be attributed an alleviated legal responsibility. By contrast, if the agent is unlucky, then her responsibility is to be attributed to the full extent and the renunciation action does not matter. In other words, the law holds agents responsible for outcomes which are not under their control. A competing account of responsibility is the Kant-oriented account. This account states that people should not be held morally and legally responsible for outcomes which are not under their control.

During a courtroom hearing, when analyzing the case at stake, judges or juries are only presented either with successful or unsuccessful renunciation cases. They never see both at once and, as argued in the introduction, there is no jury instruction that would constitute a good incentive to compare the two counterfactual cases. For this reason, the consequence-oriented formulation of the legal rules is not necessarily the result of a careful, all things considered, consideration of the principles that should guide agent’s responsibility for criminal outcomes.

Since the present experimental results point out to the fact that, when presented with both the lucky and unlucky cases at once, participants do not assess the responsibility of the two agents differently, perhaps the content of the legal rules does not really reflect what, upon reflective judgment we perceive as fair and just. The genuine, reflective folk concept of legal responsibility expressed in the within-subjects design is Kant-oriented rather than consequence-oriented.

If this is the case, perhaps we should reconsider the rationale of the legal rules, or at least of the jury instructions, so as to make them less dependent on outcomes. In other words, the legal rules should not adopt merely consequence-oriented approaches, but rather a Kant-oriented equal responsibility only for factors which are under the agents’ control. There can be other practical reasons for upholding the current state of the renunciation defense. My claim is modest – even folk intuitions do not support a pure consequence-oriented approach and thus perhaps it is yet an additional argument toward a more Kant-oriented direction in the construction of the renunciation defense could perhaps be a step worth considering (even if by itself it is not conclusive).

Moreover as to practical considerations, as Gabriel Mendlow, in his 2019 paper argues, the interpretation of criminal legal rules (especially rules pertaining to punishment) is a special domain. Consequently, a good deal of considerations pertaining to punishment ascriptions are beyond the semantic meaning of the legal rules, which is especially pertinent in common law systems. In these systems it is the interpretive judicial practice which plays an important role: ‘The wrong a law criminalizes turns out to be the product of diverse factors, including not only what the law says, but also how legal officials exercise their discretion to charge, convict, and sentence’ (Mendlow, 2019, p. 107). Survey experiments can shed more light on such practice (Adams & Steadman, 2004; Pirker & Skoczeń, 2022a, 2022b; Pirker & Smolka, 2019; Sarin et al., 2020).

What strengthens my case against the current construction of the renunciation defense is the general rule of penal codes around the world which takes the mental state assessment to be the same independently of the luck-dependent outcome of an action.

Interestingly, the Model Penal Code usually punishes attempts and perpetration in an equal manner (Dressler, 2012). Analogously, the Polish penal code states in article 14 par 1 that: ‘The court shall impose a penalty for an attempt within the limits of the penalty provided for the given offence.’ However, the German penal code states in its article 23 par 2 that: ‘A more lenient penalty may be imposed for an attempt than for a completed offence’. So perhaps, and this remains an avenue for further studies, the German code captures the folk intuition that punishment is to be differentiated more than culpability, perhaps due to its pragmatic functions. Nevertheless, even if differences in punishment would be to remain, if an agent were to be granted the renunciation defense similarly in both the lucky and unlucky cases, this would not go against folk intuitions of fairness. Moreover, it would be in line with the criminal law’s distaste for strict liability.

### Limitations

There are several limitations of the present study that will require further future investigation.

The first limitation that remains a prospect for future inquiry is the role of causal judgments in the renunciation cases (Knobe & Shapiro, 2021; Prochownik, 2022). Outcome luck is also causal luck: whether the initial causal chain breaks and whether the criminal outcome occurs is not under the agent’s control. Future studies should investigate to what extent is the relation between outcome and blame expressed through causal judgments as well as ascriptions of mental states (cf. Güver & Kneer, 2022; Zehnder & Kneer, in preparation).

The second limitation concerns the language of criminal law. Namely, the consequence-oriented terminology is deeply ingrained in the terms which have been used in criminal law for centuries. As Joel Feinberg argues, if murder and attempted murder are to be considered on a par, then, without terminological alterations, ‘participants in the criminal process would start saying some very odd things, such as “Jones murdered Smith although Smith is still alive”’ (Feinberg, 2003, p. 79).

Since reforming legal rules is usually a difficult and controversial endeavor due to manifold additional practical reasons that can influence the process, perhaps just a system of judicial and jury training could be a future direction. The aim of the training would be to uncover the genuine Kant-oriented intuitions hidden behind the dangerously seductive routine of centuries old criminal law language and case law replete with consequence-oriented judgments.

The folk intuitions captured in the present experiments show that the control in the first instance (administering poison) does cover to a certain extent the post-attempt effort at (unsuccessful) renunciation. However, there still is a normative difference in assessment of someone who merely perpetrates and someone who perpetrates and (unsuccessfully) renunciates. If we allow pure luck to decide in the second case and preclude a difference between a perpetrator and a perpetrator who (unsuccessfully) renounced her criminal intent, then there is a risk that an important normative distinction disappears. In sum, I do not want to argue that renunciation wipes out any traces of initial criminal intent, but only that it changes the overall assessment of the agent.

A similar line of argument is developed by Alex Sarch, 2019. Sarch argues with respect to willful ignorance that the lack of *mens rea* is irrelevant and is erased by the agent’s conscious actions/omissions in order to deprive oneself from knowledge of criminal facts (for an empirical study of willful ignorance, which finds that participants nevertheless attribute less knowledge to a willfully ignorant agent as compared to a knowing perpetrator see Kirfel & Hannikainen, 2022). Perhaps in renunciation cases, even if the antidote was super effective, the offender should know that she administered the poison in the first place and thus she should know that she created a danger in the first place. In other words, perhaps the perpetrator should be aware that the initial intent cannot be completely erased, however, the renunciation act should not be ignored by the criminal legal system either.

James Edwards & Andrew Simester argue that, broadly speaking, outcomes should be taken into account in the definition of offences because this enables to communicate to society that different wrongs have been committed when there is an actual death resulting from a criminal action, as compared to the situation when there is no outcome (for instance as a result of an attempted murder) (Edwards & Simester, 2019). I agree that, traditionally, the communicative function of criminal law is important. Criminal law can be an important tool to communicate to society what is a wrong which should not be committed. However, I think that this is not the only way of conveying to society which behaviors are wrong, even if it is a traditional way of doing so. Moreover, participants assessed the wrongness of the protagonist’s action similarly both in between and within subjects’ design, so the intuition that different wrongs are committed in an attempt and perpetration case was not shared by the experimental participants.

By contrast, maintaining different descriptions/labeling (perpetration *versus* attempt for instance) for different wrongs resulting out of the same *mens rea* and action implicates or at least suggests that these different wrongs merit different levels of blame and punishment, which, I agree with Edwards & Simester, should not be the case. After all, as Edwards & Simester argue, there is merely a correlation, but certainly not an automatic causation relation, between outcomes and *mens rea*s (Edwards & Simester, 2019). In other words, two sorts of considerations can be at play. First, since we live in a world constrained by our limited epistemic access to facts, we are always constrained by what can and what cannot be proved in court. I agree that these considerations shape to a large extent the current mechanics of the criminal procedural system. However, it is not clear to what extent should the evidentiary constraints shape the second type of considerations, namely considerations of what principles of responsibility reflect best our views of what is fair and just.

Thus, I believe that the switch of vocabulary could be a step toward signaling society that blaming and punishing attempters and perpetrators at similar levels is reflected in our shared ethical principles.

## Conclusion

Survey experiments within the domain of moral psychology depict that the differentiation of responsibility ascriptions based solely on the outcome of an action which is outside of the agent’s control is not the result of careful, all things considered, moral judgment but rather the result of a quick, unreflective reaction to the severity of an outcome. When reflective, study participants agree that responsibility should not depend on factors beyond the agent’s control. I argue that this finding has a direct application to the legal domain. If an agent undertakes a criminal action and, on second thoughts, tries to undo the action, the final result can be a matter of sheer causal luck: it is not under the agent’s control. The renunciation defense that enables to mitigate punishment for active repentance of the criminal action is available only to agent whose action does luckily not lead to a criminal result. By contrast, the unlucky agent who undertakes exactly the same action in the same circumstances cannot resort to the defense. This consequence-oriented formulation of the legal rules on the renunciation defense is not the result of a careful building upon our shared principles of just and fair responsibility such as the assessment of a mental state independently of the outcome of an action. When reflective, that is presented with the relevant alternative outcome, the folk concept of responsibility desists from consequence-oriented ideas: it becomes more Kant-oriented.

## Supplementary Information

Below is the link to the electronic supplementary material.

Supplementary file1 (DOCX 26 kb)

## Footnotes

1 *United States v. U.S. Gypsum Co*., 438 U.S. 422, 438 (1978).

2 *Morissette v.United States*, 342 U.S. 246, 250 (1952).

3 The law governing workmen's compensation, abnormal or ultrahazardous activities, nuisance, and products liability (Coleman, 2002).

4 The same would apply in France, Germany, Japan, Switzerland and the UK. The Polish criminal code allows for a mitigation of punishment with unsuccessful active repentance, yet case law suggests that these rules are rarely applied.

5 cf. People v. Graham 176 App. Div. 38 (N.Y. App. Div. 1916); Munford v. State, 923 N.E.2d 11 (Ind. Ct. App. 2010), Licciardello v. New Jersey 2017, New Hampshire v. Brown 2010, Michigan v. Crawford 1998, Riley v. Semple 2018, Sisselman v. New York 1989.

6 Sheckles v. State, 501 N.E.2d 1053, 1055 (Ind. 1986); Barnes v. State, 378 N.E.2d 839, 843 (Ind. 1978); State v. Eagle, 611 P.2d 1211, 1213 (Utah 1980). People v. Collins, 137 N.E. 753 (NY 1922) Steven Burke HARRIMAN, Appellant, v. STATE of Florida, Appellee. No. 1D14-2147.Decided: August 24, 2015), Louis v. Texas 1998, Pennsylvania v. Schofield 2019, O’Neil v. Texas 2006, Dworkin v. US 2015, Connecticut v. Richardson 1996, Johnston v. New York, 1982, New Jersey v. Daniels 2013.

7 Japan (art 43 Japanese Penal Code) and Switzerland (art 22 and 23 of the Swiss Penal Code, cf cases 6B_1140/2018 and 6B_840/2017).

8 Consider a case from Kraków in Poland (II AKa 65/16), where the facts were the following: A hated B. B was walking on foot and behaved in a provocative way. A was drunk and decided to run B over with his car, yet at 20 m to B, A changed his mind and turned. Unfortunately, it was too late, the car slipped, and B was injured yet not killed. The court of first instance ruled that A did not commit a murder attempt because of his renunciation of intent to murder. A was ascribed only an intentional effect of bodily injury. However, the prosecution appealed and the court of second instance overturned the judgment of the first instance. The second instance ruled that renunciation was not successful because at 20 m to B, A had no control over the effect. As a result, the court ruled that A committed attempted murder and that A did not renounce his criminal intent. Moreover, the court charged A with an intentional effect of bodily injury, which, combined with the attempted murder charge, resulted in a much higher punishment. Thus, the judgement was driven by severity of harm rather than the criteria inscribed in the legal rules on criminal responsibility: the mental state of the perpetrator (*mens rea*) and his actions (*actus reus*).

9 Analogously for instance in Poland and Switzerland.

10 I do not use ‘consequence-oriented’ in the utilitarian sense. I define it as a doctrine which bases criminal responsibility on the consequences of one’s action, rather than on the perpetrator’s mental states and her actions.

11 Proving a genuine, internal change of heart is a challenging endeavor vulnerable to a multitude of potential mistakes, so in this sense it becomes closer to strict liability, if the proving of the change of heart is being neglected in practice due to probative difficulties or uncertainties. However, it is similar only in this narrow and imperfect sense, as indeed the legal rules on renunciation require that renunciation be ‘voluntary’ – thus there is need to prove (or at least try to prove) a mental state element.

12 The risk & probability problem is a problem present not only in the renunciation defense, but also in the intoxication defense as well as general intent crimes (Dimock, 2011; Mendlow, 2019). This is because, generally speaking, the risk that one will commit an offense after alcohol consumption is extremely low. An incredibly small percentage of crimes are committed after alcohol consumption. Moreover, just as in the renunciation case, there is not really a *mens rea* of the perpetrator at the moment in which the criminal outcome occurs. This is because, when intoxicated, the offender does not have control of her deeds and on the outcome of her actions. As the mentioned Authors argue, a similar case arises with general intent crimes in which intention can be ascribed for an extremely broad range of consequences and it is extremely controversial whether the accused indeed foresaw these consequences. Finally, it is extremely controversial whether the probability of these consequences occurring was elevated to a level that would license intent ascriptions in general. For similar issues in the motives ascriptions cf. (Morse, 2004; Spranca et al., 1991).

13 Angela Smith argues that, descriptively speaking, we have a rationalist rather than volitionist account of responsibility—we sometimes hold people responsible for things which are outside of their control and it is attitudes and emotions that are decisive. However, I try to argue with my empirical data, that such moral luck endorsement intuition is substantially mitigated in a within subjects’ design (Smith, 2005; Smith, 2008).

14 There is a difference between cases of an incomplete attempt, which might even be merely some sort of formulation of criminal plans, close to mere criminal thought as well as a completed attempt (Mendlow, 2021). Moreover, there is also a difference between a completed attempt as well as a completed attempt with a renunciation of criminal intent. Since there is a normative difference of degree between these cases, I limit the scope of interest in the paper to merely the tested cases, which are cases where the difference between successful and unsuccessful renunciation is based on luck. Therefore, at the same time, luck also provides here for the difference between an attempted and committed crime.

15 What could hint toward this hypothesis is a survey study of renunciation cases in which participants were found to be biased by religion in their assessments of how likely an agent is to renounce the criminal endeavor. Namely, if participants were told that the protagonist is a Christian, then they answered that he was more likely to renounce criminal intent than when they were told that the protagonist is Muslim (Sood, 2019).

16 Studies 1 and 2 were preregistered and run together so as to ensure that no participant would take the study twice. Link to preregistration: https://aspredicted.org/ji3ys.pdf; all data, preregistrations, appendix and source files can be found in the repository: https://osf.io/r5u4b/.

17 Studies 1 and 2 were preregistered and run together so as to ensure that no participants would take the study twice. Link to preregistration: https://aspredicted.org/ji3ys.pdf.

18 Link to preregistration: https://aspredicted.org/h9h7a.pdf.
