# Supplementary material for: Renouncing the attempt versus perpetration distinction
Source: Synthese. 2023 Jan 11;201(1):26. doi: 10.1007/s11229-022-04000-6 (PMC9834164; doi:10.1007/s11229-022-04000-6)
Supplement: Supplementary file 1 — Supplementary file1 (DOCX 26 kb) [file 11229_2022_4000_MOESM1_ESM.docx]

# Appendix

## A1 Further details regarding experiments: study 1 (between subjects); study 2 (within subjects) and study 3 (contrastive design)

### A 1.1 Further details concerning study 2

#### A 1.1.1. Order effects

In a series of independent samples t-tests, I explored potential order effects for all DVs in each condition. No evidence could be found for order effects (all *ps >*.170, two-tailed), cf. Table A1.

|  | t (78) | p | 95% CI | |
| --- | --- | --- | --- | --- |
|  |  |  | Lower | Upper |
| Wrong good | 1.39 | .170 | -.20 | 1.10 |
| Wrong bad | 1.04 | .301 | -.26 | .83 |
| Blame good | 1.30 | .198 | -.22 | 1.05 |
| Blame bad | .09 | .925 | -.46 | .50 |
| Punishment good | .31 | .758 | -.58 | .79 |
| Punishment bad | .32 | .752 | -.53 | .73 |

**Table A1** Order effects on wrongness, blame, and punishment judgments in a within-subjects design: Independent samples t-test (t-test for Equality of Means).

### A 1.2 Further details regarding experiment 3 (contrastive design)

#### A 1.2.1 Mean difference from endpoint and midpoint:

|  | difference from endpoint | | | | | | difference from midpoint | | | | |
| --- | --- | --- | --- | --- | --- | --- | --- | --- | --- | --- | --- |
|  | M | SD | t(102) | p | 95% CI | d | t(102) | p | 95% CI | d |  |
| Wrongness | 6.27 | 1.44 | -5.12 | <.001 | [-1.01;-.45] | .72 | 15.98 | <.001 | [1.99;2.55] | 2.23 |  |
| Blame | 6.29 | 1.39 | -5.17 | <.001 | [-.98;-.44] | .72 | 16.72 | <.001 | [2.02;2.56] | 2.33 |  |
| Punishment | 6.00 | 1.54 | -6.59 | <.001 | [-1.30;-.70] | .92 | 13.18 | <.001 | [1.70;2.30] | 1.84 |  |

Table A2: Mean difference from endpoint 7 and midpoint 4; 95% confidence intervals are given for the mean difference.

#### A 1.2.2. abstract comparative task:

In the abstract comparative task, A one-way repeated-measures ANOVA determined that participants’ mean answers to the wrongness, blame, and punishment questions differed significantly (F(2,204) = 5.98 , p = .003 , η2 = .055).

Participants disagreed more with the claim that the morally lucky and morally unlucky agents deserved the same punishment than they disagreed with the claims that their actions were equally wrong and equally blameworthy, and I did not find any evidence that they responded differently to these two latter claims. The means for all three measures were significantly above the neutral midpoint 4 and significantly below the endpoint 7 (complete agreement).

Following Lench et al., 2015, I calculated the percentage of participants who agreed with the claim that the two agents should be judged identically. I also aggregated the number of participants who responded with “completely agree” (Likert scale endpoint 7). The results, presented in Table A3, were consistent with the findings from Study 2: For wrongness and blame the large majority of participants (over 87%) agreed that the two agents should be judged identically (Likert scale > 4). Over half of the participants chose the endpoint of the Likert scale. As regards punishment, slightly less, 82% of the participants agreed that the two agents deserve the same punishment, and over a half completely agreed with an assessment of this sort.

| Measure | Wrongness | Blame | Punishment |
| --- | --- | --- | --- |
| Likert Scale >4 | 87% | 89% | 82% |
| Endpoint 7 | 68% | 70% | 58% |

Table A3 Proportions of participants who agreed (Likert scale > 4) or completely agreed (endpoint 7) that the two agents should be judged identically with respect to wrongness, blame, and punishment.

#### A 1.2.3 Mean difference from endpoint and midpoint (abstract comparative task):

|  |  | | | Difference from endpoint | | | Difference from midpoint | | |
| --- | --- | --- | --- | --- | --- | --- | --- | --- | --- |
|  | M | SD | t(102) | | p | 95% CI | t(102) | p | 95% CI |
| Wrongness | 6.29 | 1.32 | -5.46 | | <.001 | [-.97;-.45] | 17.64 | <.001 | [2.03;2.55] |
| Blame | 6.29 | 1.33 | -5.43 | | <.001 | [-.97;-.45] | 17.54 | <.001 | [2.03;2.55] |
| Punishment | 5.97 | 1.58 | -6.61 | | <.001 | [-1.34;-.72] | 12.65 | <.001 | [1.66;2.28] |

Table A4: Mean difference from endpoint 7 and midpoint 4 for abstract comparative task; 95% confidence intervals are given for the mean difference.
